# Delphi case: Sharing of clinical experiences for improvement in the treatment of chronic venous disease

**DOI:** 10.3389/fcvm.2022.921235

**Published:** 2022-07-18

**Authors:** Giuseppe Camporese, Teresa Lucia Aloi, Angelo Santoliquido

**Affiliations:** ^1^General Medicine Unit, Thrombotic and Haemorrhagic Disorders Unit, Department of Internal Medicine, University Hospital of Padua, Padua, Italy; ^2^Cardio-Angiology Unit of Montescano and Pavia Institute, Istituti Clinici Scientifici Maugeri Istituto di Ricerca e Cura a Carattere Scientifico (IRCCS), Pavia, Italy; ^3^Unit of Angiology, Cardiovascular Department, Catholic University of Sacro Cuore, Gemelli Policlinic Foundation—IRCCS, Rome, Italy

**Keywords:** chronic venous disease, Delphi Consensus, varicose veins, therapeutic management, CVD management

## Abstract

Chronic venous disease (CVD) is a common condition with major health consequences that is associated with poor long-term prognosis, significant socioeconomic impact, disabling symptoms, and reduced quality of life. To provide a novel evidence-based approach in the management of CVD, a consensus process (“Delphi Case”) following a first Delphi Consensus was conceived. With a real-life fashion analysis, a steering committee formed by 3 expert leaders on chronic venous disease drove a panel of 77 expert Italian angiologists/vascular surgeons along a collegial discussion, integrating data coming from the guidelines recommendations of different Vascular Scientific Societies with the consensus agreement statements gathered from the first Delphi Consensus, and with data coming from the discussion of few statements in which there was disagreement. From July 15 to October 16, 2020, demographic, anamnestic, objective, and therapeutic data coming from a total of 2,275 patients were collected by the experts panel using a predefined case report form. The results of this second consensus provided a real-life picture of CVD management in the Italian population and clearly showed that a tailored therapeutic approach together with an appropriate lifestyle (e.g., diet, physical activity, weight loss) must be considered as the milestones for the CVD-related signs and symptoms clinical improvement in daily clinical practice. An evaluation of the adherence and of the efficacy of the prescribed pharmacological and compressive treatment in a medium-long term follow-up of the study population has been planned as the last step of this course and will be object of a future final publication.

## Introduction

Chronic venous disease (CVD) is major health and social problem in western countries. Its prevalence ranges from 5 to 30% in the adult population (1–3). Prevalence rates for varicose veins are even much higher, reaching 73% in women and 56% in men (1–3). The clinical picture of CVD typically includes worsening limb heaviness, pain and/or edema. In time, it can progress to its most feared consequence; that is, venous ulcer, whose prevalence is 1–2% in all CVD patients, topping to 4% in patients aged over 80 years.

For a correct definition of CVD, international guidelines recommend the use of the Clinical, Etiologic, Anatomic, Pathophysiologic (CEAP) classification, that allows for an accurate assessment of the disease burden. The CEAP classification grants a precise staging and a correct grading of the CVD patient, defining both the clinical severity level and the evolution of the disease in an objective and reproducible fashion.

The diagnosis of CVD underpins multiple diagnostic and therapeutic questions, and several scientific Societies of Vascular Medicine and Surgery have issued both national and international guidelines about its management; nevertheless, all recommendations focused on the treatment of the diseased population (1, 4–11).

In 2019, aiming to provide clinicians with practical guidance and to integrate national and international guidelines, we conceived a Delphi Consensus suggesting some personalized diagnostic-therapeutic approaches to CVD (12). In that previous essay a Steering Committee of five experts formulated 24 statements, divided into the main areas of diagnosis and therapeutic management. A panel of 28 angiologists/vascular surgeons, selected across Italy on the basis of their interest and expertise in the management of patients with CVD, was invited to vote these statements and to participate in the Delphi process (12). The entire process took place in ~8 months and a first online statement vote followed by a final consensus meeting were planned, where the expert panel and Steering Committee discussed the results of the online consensus survey followed by another round of voting for statements with partial consensus or negative consensus. At the end of this first Delphi process a broad consensus was reached on 22 out of 24 statements initially formulated by the Steering Committee.

This new consensus process (“Delphi Case”), aims to integrate the recommendations of several scientific Societies with the statements agreed upon during the mentioned Delphi Consensus. In particular, the latter was improved though the revision the only two statements on which the panel did not previously agree; furthermore, it was integrated with real-life data coming from patients directly managed by the panel.

## Materials and methods

The Delphi process is a widespread, rapid, and convenient method to reach expert consensus on specific issues. The process built on successive iterations, in a survey type format. On each voting round, participants assess the results, give feedback, and subsequently modify a statement or recommendation, until a broader consensus is reached (13). The Delphi approach combines the principles of evidence-based medicine, supported by systematic literature review, with an iterative and anonymous voting process. Such a process overcomes many issues associated with group-dynamics in decision-making committees, as the experts can thus provide their opinions freely, individually and anonymously (14).

For this Delphi Case we used a modified two-stage Delphi technique, omitting the qualitative rounds, because we built on the statements derived from the former Delphi Consensus (15). The Delphi Case took place from July 15 to October 16, 2020.

The Steering Committee comprised 3 experts (GC, TA, AS), involved in the first Delphi Steering Committee, identified on several criteria, including their expertise and/or academic rank, number publications, attendance at national and international meetings, and participation in clinical trials.

The list of statements from the Delphi Consensus was substituted with an observational case-report form (CRF), drawn up by the Steering Committee to allow for collecting real-life demographic, historical, objective, and therapeutic data about patients with CVD. The CRF was shared with a panel of 77 angiologists/vascular surgeons from selected vascular centers across Italy (see Supplementary Material for the list of co-authors and for the CRF), including 28 experts who had participated to the first Delphi Consensus, and another 49 professionals, identified by the Steering Committee based on their interest and expertise in the management of patients with CVD. Panelists were adequately informed about the design, the aim and the results of the first Delphi Consensus, and about the aim of the Delphi Case.

Panelists were asked to anonymously fill in the CRFs with data of their own patients with CVD. In particular, the presence or absence of telangiectasias, reticular or varicose veins, dermatitis, eczema, atrofic blanche, or venous ulcers was collected by means of a standardized physical evaluation [based on the CEAP classification (C1–C6 classes, C0 excluded)], including the number and size of venous ulcers. The intensity of symptoms (heaviness, pain, cramps, itching, paraesthesia) was assessed by means of Numeric Rating/Visual Analog Scale (NRS/VAS), ranging from 0 (absence of the symptom) to 10 (the worst intensity ever) (16, 17).

To avoid potential inclusion of patients with venous-like diseases patients assigned to C0 (zero) “C” of CEAP grading were excluded, just like it was done for the Delphi Consensus; therefore, only patients with clear signs and/or symptoms of CVD, that is, belonging to C1–C6 CEAP stages, were considered for the analysis. Moreover, it must be emphasized that in this study we used the former version of the CEAP classification, because it was started before the publication most recent version (18).

## Results

Demographic characteristics of the study population are summarized in Table 1.

**Table 1:** Demographic characteristics of the study population.

**Variable**	**Sample size**	**Percentage (%)**
Gender		
Female	1,558	68.5
Male	717	31.5
Age (y)		
<44	494	21.7
44–64	846	37.2
65–94	935	41.1
Body mass index (kg*/*m^2^)		
<18.5	75	3.3
18.5–25	904	39.7
25–30	863	38.0
30–35	301	13.2
35–40	88	3.8
≥40	44	2.0
Family history of CVD	1,767	77.7
Family history of VTE	494	21.7
Personal history of DVT	255	11.2
Personal history of SVT	662	29.1
≥1 previous pregnancy	1,183	52.0

*CVD, Chronic venous disease; VTE, Venous thromboembolism; DVT, Deep vein thrombosis; SVT, Superficial vein thrombosis*.

Overall, 2,275 patients (68.5% female, 31.5% male) were included. The mean age was 58 (SD ± 17.1) years, the age groups 44–64 years (37.2%), and 65–94 years (41.1%), being the most represented. A family history of CVD or of venous thromboembolism was recorded in 77.7 and 21.7% of the patients, respectively. A personal history of deep vein thrombosis, with or without pulmonary embolism, was recorded in 11.2% of patients, while one or more occurrences of superficial vein thrombosis was reported by 29.1%.

Almost 20% of female patients were on hormonal treatment (5.8% replacement therapy, 13.8% oral contraceptives), and 52% of women had had at least one pregnancy.

Weight and body-mass index significantly correlated with severity and progression of CVD.

Daily physical activity was considered a protective factor, because 68.5% of patients with CVD reported sedentary lifestyle together with a prolonged standing position during working activities. Overall, a NRS/VAS score ≥4/10 was significantly related to CVD; namely, 77% of patients complaining of heaviness had a score ≥4, as well as 48% of those with pain, 46% of those with muscle cramps, 36% of those with itching, and 31% of patients with paresthesia.

Concerning the objective evaluation [based on the CEAP classification, telangiectasias and spider veins were present in 82% of patients, varicose veins in 70%, edema in 49%, pigmentation in 33.4%, and eczema in 23% of patients. More advanced stages of CVD such as lipodermatosclerosis or atrophic blanche were present in 10% overall, and in 12% in patients with venous ulcers.

Only 36 and 26% of patients were using venoactive drugs and graduated compression stockings before the study; and the respective figures rose to 92 and 87% afterwards.

## Discussion

CVD is a common disease associated with both poor long-term prognosis and significant socio-economic burden; in particular, CVD leads to severe disability and reduced quality of life, especially in the advanced stages of the disease (4, 19).

For the correct definition of CVD International Guidelines recommend the use of CEAP classification, that accurately and objectively allow to assess the disease on four parameters: clinic, etiology, anatomy, and pathogenesis (4–11). This allows a precise stadiation and a correct grading of the CVD patient defining in an objective and reproducible fashion the clinical severity level and the evolution phase of the disease. This Delphi Case is a wide observational survey on the Italian population with CVD, aiming to provide practical guidelines about the diagnosis and the management of CVD (12). Our study presents for the first time the results of a large-sample retrospective survey of patients with CVD coming from all Italian regions, who were evaluated by skilled angiologists or vascular surgeons.

In our study, patients aged >44 years were mostly represented (78.3%). We observed a direct correlation between mean age, mean BMI, and CEAP stages (Table 2). Namely, we recorded a progressive increase in the mean age and mean BMI (433 patients with a BMI > 30) with increasing CEAP classes. In the mild and moderate CEAP stages (C1–C3) female patients were more frequent, whereas in the severe (C4–C6), male and female patients were equally represented. Our findings partially agree with the data reported by the Bonn Vein Study, in which the risk of developing CVD was increased in elderly and obese female patients (20).

**Table 2:** Correlation between BMI, age, and CEAP class in the study population.

**CEAP (class)**	**BMI (mean)**	**Age (y) (mean)**
1	24.79	45
2	25.90	54
3	26.60	59
4	25.74	63
5	26.86	67
6	28.70	70

*BMI, Body mass index; CEAP, Clinical, etiologic, anatomic, pathophysiologic classification*.

Every patient included in the analysis was first labeled as symptomatic or asymptomatic. Then, in symptomatic patients, the intensity of complaints was systematically evaluated by the NRS/VAS scales. In our study population some 20% of patients were asymptomatic. Moderate to severe (4–10) VAS/NRS scores were recorded in three quarters of patients complaining of leg heaviness, in almost half of subjects reporting leg pain or muscle cramps, and in one third of those with itching or paresthesia. Such findings are in line with the epidemiological and observational study on CVD of the “Vein Consult Program” (21).

CVD symptoms, mainly pain, are strictly related to the inflammation of the venous wall, that could be present even in the early stages of CVD (C1 patients) (22); counteracting inflammation with specific drugs, instead of only working at the symptomatic level with painkillers, could prevent the progression to severe stages of disease. Also, pain and infections negatively impact on the quality of life of CVD patients; particularly, among other symptoms of CVD, pain is the main driver of urgent vascular visits (23, 24). Of note, the onset of venous symptoms predicts the worsening of CVD in the long-term (25).

Although leg symptoms were present in more than 80% of the patients, only roughly one third was on venoactive drugs, and one fourth wore graduated elastic stockings. We must emphasize that after the visit of a Vascular Specialist the prescription of venoactive drugs and graduated compression stockings rose 3-fold. This clearly shows the importance of being taken in charge by a vascular specialist (6, 26–30).

Another intriguing finding of our study concerns the frequency of superficial vein thrombosis (SVT) traditionally considered a self-limiting, benign disease (31). Such view recently changed, and our findings confirm this new line. Surprisingly, almost 30% of our patients had a history of SVT, a figure that is higher than what would be expected based on the natural course of the disease. This could be partially explained by the prolonged lockdown due to the SARS-CoV2 pandemic, during which several concurring situations, including reduced physical activity, increased body weight, reduced access to control visits, could have played a causative role, especially in advanced stages of the disease (C4–C6). On the other side, we must consider that this was an historical finding, that does not necessarily imply an instrumental diagnosis of SVT; and this should indeed be regarded as a limitation of our study.

Other limitations are as follows: first, no follow-up data is currently available for patients. However, we are planning a further retrospective analysis concerning the clinical evolution of the same study population, together with an analysis of the adherence and the efficacy of the prescribed pharmacological and compression treatment. Second, data about the instrumental objective diagnosis of CVD was not recorded.

## Conclusions

Our results are noteworthy. A large patient sample, exclusively evaluated by Vascular Specialists, provides a real-life picture of CVD in Italy. We believe that a more structured management of CVD would be desirable, to prevent psycho-physical disability, which the inadequately treated patient may encounter, with impaired quality of life and considerable socio-economic implications. In this sense, a tailored early pharmacologic and/or compression therapy are the milestones of the management of CVD, targeting the pathogenetic mechanisms (e.g., inflammation, wall damage) which are the basis of the development of the disease. Such a therapeutic approach, together with an appropriate lifestyle (e.g., diet, physical activity, weight loss) could lead to a clinical improvement of CVD-related signs and symptoms.

## Data availability statement

The original contributions presented in the study are included in the article/Supplementary Material, further inquiries can be directed to the corresponding author.

## Ethics statement

Ethical review and approval was not required for this study in accordance with the local legislation and institutional requirements.

## Author contributions

All authors listed have made a substantial, direct, and intellectual contribution to the work and approved it for publication.

## Funding

This study received an unrestricted educational grant from Neopharmed Gentili, Italy. The funder was not involved in the study design, collection, analysis, interpretation of data, the writing of this article, or the decision to submit it for publication.

## Conflict of interest

The authors declare that the research was conducted in the absence of any commercial or financial relationships that could be construed as a potential conflict of interest.

## Publisher's note

All claims expressed in this article are solely those of the authors and do not necessarily represent those of their affiliated organizations, or those of the publisher, the editors and the reviewers. Any product that may be evaluated in this article, or claim that may be made by its manufacturer, is not guaranteed or endorsed by the publisher.

## References

[1] Beebe-Dimmer JL Pfeifer JR Engle JS Schottenfeld D. The epidemiology of chronic venous insufficiency and varicose veins. Ann Epidemiol. (2005) 15:175–84. 10.1016/j.annepidem.2004.05.015 15723761

[2] Evans CJ Fowkes FG Ruckley CV Lee AJ. Prevalence of varicose veins and chronic venous insufficiency in men and women in the general population: Edinburgh vein study. J Epidemiol Community Health. (1999) 53:149–53. 10.1136/jech.53.3.149 10396491 PMC1756838

[3] Fowkes FG Evans CJ Lee AJ. Prevalence and risk factors of chronic venous insufficiency. Angiology. (2001) 52 (Suppl. 1):S5–15. 10.1177/0003319701052001S02 11510598

[4] Eberhardt RT Raffetto JD. Chronic venous insufficiency. Circulation. (2014) 130:333–46. 10.1161/CIRCULATIONAHA.113.006898 25047584

[5] Mansilha A Sousa J. Pathophysiological mechanisms of chronic venous disease and implications for venoactive drug therapy. Int J Mol Sci. (2018) 19:1669. 10.3390/ijms19061669 29874834 PMC6032391

[6] Nicolaides A Kakkos S Baekgaard N Comerota A de Maeseneer M Eklof B. Management of chronic venous disorders of the lower limbs. Guidelines according to scientific evidence part I. Int Angiol. (2018) 37:181–254. 10.23736/S0392-9590.18.03999-8 29871479

[7] Robertson L Lee AJ Evans CJ Boghossian S Allan PL Ruckley CV. Incidence of chronic venous disease in the Edinburgh vein study. J Vasc Surg Venous Lymphat Disord. (2013) 1:59–67. 10.1016/j.jvsv.2012.05.006 26993896

[8] National Institute for Health Care Excellence. Varicose Veins in the Legs: The Diagnosis and Management of Varicose Veins. NICE. Available online at: www.nice.org.uk/cg168 25535637

[9] Gloviczki P Comerota AJ Dalsing MC Eklof BG Gillespie DL Gloviczki ML. The care of patients with varicose veins and associated chronic venous diseases: clinical practice guidelines of the society for vascular surgery and the American venous forum. J Vasc Surg. (2011) 53 (5 Suppl):2S−48S. 10.1016/j.jvs.2011.01.079 21536172

[10] Wittens C Davies AH Baekgaard N Broholm R Cavezzi A Chastanet S. Editor's choice - management of chronic venous disease. Clinical practice guidelines of the European society for vascular surgery (ESVS). Eur J Vasc Endovasc Surg. (2015) 49:678–737. 10.1016/j.ejvs.2015.02.007 25920631

[11] Agus GB Allegra C Arpaia G De Franciscis S Gasbarro V. Linee Guida. Collegio Italiano di Flebologia. Revisione 2013. Acta Phlebol. (2013) 14 (2 Suppl. 1):1–160.

[12] Aloi TL Camporese G Izzo M Kontothanassis D Santoliquido A. Refining diagnosis and management of chronic venous disease: outcomes of a modified Delphi consensus process. Eur J Intern Med. (2019) 65:78–85. 10.1016/j.ejim.2019.03.005 30898385

[13] Loblaw DA Prestrud AA Somerfield MR Oliver TK Brouwers MC Nam RK. American society of clinical oncology clinical practice guidelines: formal systematic review-based consensus methodology. J Clin Oncol. (2012) 30:3136–40. 10.1200/JCO.2012.42.0489 22778311

[14] Bellizzi V Bianchi S Bolasco P Brunori G Cupisti A Gambaro G. A Delphi consensus panel on nutritional therapy in chronic kidney disease. J Nephrol. (2016) 29:593–602. 10.1007/s40620-016-0323-4 27324914

[15] Stewart D Gibson-Smith K MacLure K Mair A Alonso A Codina C. A modified Delphi study to determine the level of consensus across the European union on the structures, processes and desired outcomes of the management of polypharmacy in older people. PLoS ONE. (2017) 12:e0188348. 10.1371/journal.pone.0188348 29155870 PMC5695766

[16] Downie WW Leatham PA Rhind VM Wright V Branco JA Anderson JA. Studies with pain rating scales. Ann Rheum Dis. (1978) 37:378–81. 10.1136/ard.37.4.378 686873 PMC1000250

[17] Huskisson EC Jones J Scott PJ. Application of visual-analogue scales to the measurement of functional capacity. Rheumatol Rehabil. (1976) 15:185–7. 10.1093/rheumatology/15.3.185 968347

[18] Lurie F Passman M Meisner M Dalsing M Masuda E Welch H. The 2020 update of the CEAP classification system and reporting standards. J Vasc Surg Venous Lymphat Disord. (2020) 8:342–52. 10.1016/j.jvsv.2019.12.075 32113854

[19] Nicolaides AN. Chronic venous disease and the leukocyte-endothelium interectation: from symptoms to ulceration. Angiology. (2005) 56 (Suppl 1):11–9. 10.1177/00033197050560i103 16193221

[20] Wrona M Jockel KH Pannier F Bock E Hoffmann B Rabe E. Association of venous disorders with leg symptoms: results from the bonn vein study 1. Eur J Vasc Endovasc Surg. (2015) 50:360–7. 10.1016/j.ejvs.2015.05.013 26141786

[21] Rabe E Guex JJ Puskas A Scuderi A Fernandez Quesada F; VCP Coordinators. Epidemiology of chronic venous disorders in geographically diverse populations: results from the vein consult program. Int Angiol. (2012)31:105–15. 22466974

[22] Partsch H Flour M Coleridge Smith P Benigni JP Cornu-Thénard A Delis K. Indications for compression therapy in venous and lymphatic disease. Consensus based on experimental data and scientific evidence. Under the auspice of the IUP. Int Angiol. (2008) 27:193–219. 18506124

[23] Andreozzi GM Cordova RM Scomparin A Martini R D'Eri A Andreozzi F. Quality of life in chronic venous insufficiency. An Italian pilot study of the Triveneto region. Int Angiol. (2005) 24:272–7. 16158038

[24] Zenati N Bosson JL Blaise S Carpentier P. [Health related quality of life in chronic venous disease: systematic literature review. J Med Vasc. (2017) 42:290–30. 10.1016/j.jdmv.2017.07.001 28964388

[25] Benigni JP Bihari I Rabe E Uhl JF Partsch H Cornu-Thenard A. Venous symptoms in C0 and C1 patients: UIP consensus document. Int Angiol. (2013) 32:261–5. 23711678

[26] Kakkos SK Allaert FA. Efficacy of ruscus extract, HMC and vitamin C, constituents of Cyclo 3 fort(R), on improving individual venous symptoms and edema: a systematic review and meta-analysis of randomized double-blind placebo-controlled trials. Int Angiol. (2017) 36:93–106. 10.23736/S0392-9590.17.03815-9 28225220

[27] Tufano A Arturo C Cimino E Di Minno MN Di Capua M Cerbone AM. Mesoglycan: clinical evidences for use in vascular diseases. Int J Vasc Med. (2010) 2010:390643. 10.1155/2010/390643 21152191 PMC2989756

[28] Mariani F. (Coordinator) Consensus Conference on Compression Therapy. 2nd edition. Torino: Minerva Medica (2009).

[29] Shadrina AS Smetanina MA Sevostyanova KS Shevela AI Seliverstov EI Zakharova EA. Polymorphism of matrix metalloproteinases genes MMP1, MMP2, MMP3, and MMP7 and the risk of varicose veins of lower extremities. Bull Exp Biol Med. (2017) 163:650–4. 10.1007/s10517-017-3871-2 28944430

[30] Slonkova V Slonkova V Jr Vasku A Vasku V. Genetic predisposition for chronic venous insufficiency in several genes for matrix metalloproteinases (MMP-2, MMP9, MMP-12) and their inhibitor TIMP-2. J Eur Acad Dermatol Venereol. (2017) 31:1746–52. 10.1111/jdv.14447 28662285

[31] Wassel CL Rasmussen-Torvik LJ Callas PW Denenberg JO Durda JP Reiner AP. A genetic risk score comprising known venous thromboembolism loci is associated with chronic venous disease in a multi-ethnic cohort. Thromb Res. (2015) 136:966–7. 10.1016/j.thromres.2015.09.016 26442836 PMC4718662

