# Supplementary material for: Delphi case: Sharing of clinical experiences for improvement in the treatment of chronic venous disease
Source: Front Cardiovasc Med. 2022 Jul 18;9:921235. doi: 10.3389/fcvm.2022.921235 (PMC9339892; doi:10.3389/fcvm.2022.921235)
Supplement: Supplementary file 1 [file Data_Sheet_1.PDF]

LIST OF INVESTIGATORS

**Adamo Matteo Roberto** - *Grado (Go)*	**Giuliani Enrico** - *Viterbo*
**Alabiso Roberto** - *Gela (Cl)*	**Ivaldi Claudio** - *Acqui Terme (Al)*
**Alongi Giovanni** - *Agrigento*	**Leo Enrico** - *Lecco*
**Arboit Marco** - *Abano Terme (Pd)*	**Lombardo Almarosa** - *Palermo*
**Attilio Fabio** - *Torre del Greco (Na)*	**Luminello Felice** - *San Valentino Torio (Sa)*
**Aversano Vincenzo** - *Caserta*	**Lussardi Gian Luca** - *Marone (Bs)*
**Bandieramonte Giovanni** - *Catania*	**Maggi Giacomo Filippo** - *Milano*
**Barboni Maria Grazia** - *San Lazzaro di Savena*	**Maione Massimo** - *Aosta*
**Barone Antonio** - *Palmi (Rc)*	**Massi Marco** - *San Severino Marche (Mc)*
**Battista Antonella** - *Foggia*	**Mazzetti Matteo** - *Firenze*
**Bellandi Samuele** - *Monsummano Terme (Pt)*	**Mezzasalma Francesco** - *Milano*
**Bettini Graziano** - *Termoli (Cb)*	**Minacapelli Concetta** - *Catanzaro*
**Bianchi Pier Giovanni** - *Camino al Tagliamento (Ud)*	**Minnucci Alessandro** - *Fermo (Fm)*
**Bitossi Giorgio** - *Torino*	**Molfetta Stefano** - *Latina*
**Bucalossi Matteo** - *Siena*	**Natale Pietro** - *Pozzuoli (Na)*
**Buttazzo Francesco Giuseppe** - *Lecce*	**Nesci Antonio** - *Monterosso Calabro (Vv)*
**Cambiaso Marta** - *Genova*	**Palmas Mariella** - *Sinnai (Ca)*
**Capasso Liliana** - *Firenze*	**Pasini Pierantonio** - *Settala (Mi)*
**Catalano Francesco** - *Ruvo di Puglia (Ba)*	**Pera Maurizio** - *La Spezia*
**Cavallini Alvise** - *Verona*	**Petracca Giovanni** - *Manduria (Ta)*
**Ceccarelli Vincenzo** - *Roma*	**Piazzoni Claudia Rosa** - *Pregnana Milanese (Mi)*
**Chiodi Leonardo** - *Jesi (An)*	**Ramundo Antonio** - *Rende (Cs)*
**Coletta Silvio** - *Benevento*	**Rosi Gianluigi** - *Perugia*
**Colombani Ettore** - *Sarzana (Sp)*	**Rossi Alessandro** - *Pavia*
**Comandè Carlo** - *Monreale (Pa)*	**Rossi Mauro** - *Pavia*
**Cristaldi Carlo** - *Tremestieri Etneo (Ct)*	**Santonocito Maurizio** - *Catania*
**Cusumano Mariano** - *Alcamo (Pt)*	**Scevola Luigi** - *Vallo della Lucania (Sa)*
**D'Annibale Angelo Franco** - *Ortona (Ch)*	**Sciolla Andrea Giorgio** - *Bastia Mondovi'* (Cn)
**De Francesco Maria Maddalena** - *Matera*	**Scognamiglio Giuseppe** - *Baiano (Av)*
**De Giorgi Milco** - *Botrugno (Le)*	**SegallaArrigo** - *Costabissara (Vi)*
**De Martino Albina** - *Meta (Na)*	**Silvestro Rosario** - *Arzano (Na)*
**Di Mauro Luciano** - *Catania*	**Spiezia Stefano** - *Arzano (Na)*
**D'orazio Mirta** - *Chieti*	**Squeo Michela** - *Novara*
**Fadani Roberto** - *Leno (Bs)*	**Teutonico Paolo** - *Bologna*
**Fares Estephan** - *Fano (Pu)*	**Trani Antonio** - *Potenza*
**Filipponi Marco** - *Roma*	**Veronesi Graziella** - *Savignano sul Panaro (Mo)*
**Genovese Giovanni** - *Ispica (Rg)*	**Vitale Gerardo** - *Salerno*
**Ghilardi Gottardo** - *Calvenzano (Bg)*	**Viviani Serafino** - *Bagni di Lucca (Lu)*
**Girardi Rodolfo** - *Medesano (Pr)*	
