# Supplementary material for: Delphi case: Sharing of clinical experiences for improvement in the treatment of chronic venous disease
Source: Front Cardiovasc Med. 2022 Jul 18;9:921235. doi: 10.3389/fcvm.2022.921235 (PMC9339892; doi:10.3389/fcvm.2022.921235)
Supplement: Supplementary file 2 [file Data_Sheet_2.PDF]

DELPHI CASE

# OBSERVATION FORM

| | |
|---|---|
| DATE: | |
| GENDER SEX: | |
| AGE: | |
| WEIGHT: | |
| HEIGHT: | |
| BMI CALCULATION: | |

## PATIENT HISTORY

| | | |
|---|---|---|
| Family history of chronic venous disease | yes | no |
| Family history of venous thromboembolic disease | yes | no |
| Estrogen-progestin therapy | yes | no |
| Hormone replacement therapy | yes | no |
| Pregnancy *(if yes, how many pregnancies?)* | yes (____) | no |
| Sedentary/standing work | yes | no |
| Previous Deep Vein Thrombosis/Pulmonary Embolism | yes | no |
| Previous thrombophlebitis | yes | no |
| Previous varicophlebitis | yes | no |
| Calcium channel blocker therapy | yes | no |
| Lymphedema | yes | no |

## OBSERVATION

**Symptoms (rate on a scale of 0 to 10):**

Lower-limb heaviness: ☐

Dependent edema: ☐

Muscle cramps: ☐

Itching: ☐

Paresthesias: ☐

## STATUS AT EXAMINATION

| | | |
|---|---|---|
| Visible varicose veins | yes | no |
| Telangiectasias - reticular veins | yes | no |
| Dermatitis | yes | no |
| Eczema | yes | no |
| Atrophie blanche or white atrophy | yes | no |

Ulcers n°_____ size_________________

## CEAP CLASSIFICATION

Indicate the CEAP class with an X

| 1 | 2 | 3 | 4 | 4a | 4b | 5 | 6 |
|---|---|---|---|---|---|---|---|
| | | | | | | | |

Indicate A (asymptomatic) or S (symptomatic) with an X

| A | S |
|---|---|
| | |

## THERAPEUTIC PROTOCOL

### CURRENT PHLEBOLOGICAL TREATMENT

Pharmacological therapy yes no Which one

Elastic compression therapy yes no Which one

### PROPOSED THERAPEUTIC PROTOCOL

Pharmacological therapy yes no Which one

Elastic compression therapy yes no Which one

Surgical/Ablative Therapy yes no Which one
